# Imaging phenotype is more strongly associated with early neurological deterioration after acute isolated pontine infarction: a retrospective cohort study

**DOI:** 10.3389/fneur.2026.1913826

**Published:** 2026-10-08

**Authors:** Yanxiang Chen, Xiaoqiang Li, Hui Wang, Guonan Zhou

**Affiliations:** 1 Xiaolan Clinical Institute of Shantou University Medical College, Zhongshan, China; 2 Department of Neurology, Xiaolan People's Hospital of Zhongshan (The Fifth People's Hospital of Zhongshan), Zhongshan, Guangdong, China; 3 Department of Encephalopathy, Zhongshan Chenxinghai Hospital of Integrated Traditional Chinese and Western Medicine, Zhongshan, Guangdong, China

**Keywords:** branch atheromatous disease, early neurological deterioration, isolated pontine infarction, large artery atherosclerosis, nomogram, thrombin time, triglyceride–glucose index

## Abstract

**Background:**

Early neurological deterioration (END) is common after acute isolated pontine infarction (IPI), but the relative associations of imaging phenotype and metabolic or coagulation markers with END remain uncertain. We evaluated these domains and developed an exploratory prediction model.

**Methods:**

We retrospectively analyzed 162 patients with acute IPI. END was a ≥2-point increase in total National Institutes of Health Stroke Scale (NIHSS) score or a ≥1-point increase in NIHSS motor item 5 or 6 from the admission baseline within 7 days after admission. Imaging was modeled nominally (BAD, LAA, or SAD; SAD as reference). Primary associations were estimated by Firth logistic regression. Four prespecified Firth models were compared by AUC, and the full model underwent 1,000-resample bootstrap validation.

**Results:**

END occurred in 49 patients (30.2%). In the full Firth model, BAD (OR 20.70, 95% CI 7.10–70.04; *P* < 0.001) and LAA (OR 5.06, 95% CI 1.84–15.41; *P* = 0.001), relative to SAD, were associated with END. Thrombin time (TT) was also associated with END (OR 1.34 per second, 95% CI 1.04–1.73; *P* = 0.020); diabetes showed borderline evidence of association (*P* = 0.057), whereas TyG was not independently associated (*P* = 0.603). The apparent AUC was 0.652 (95% CI 0.562–0.742) for the base model, 0.814 (95% CI 0.745–0.883) for the base-plus-imaging model, 0.716 (95% CI 0.631–0.802) for the base-plus-metabolic/coagulation model, and 0.852 (95% CI 0.792–0.911) for the full model. Discrimination did not differ significantly between the base-plus-imaging and base-plus-metabolic/coagulation models (ΔAUC 0.098, bootstrap 95% CI −0.015–0.180; paired DeLong *P* = 0.066). All 1,000 bootstrap iterations succeeded; the optimism-corrected AUC was 0.818, with a calibration intercept of −0.023, calibration slope of 0.870, and Brier score of 0.162.

**Conclusion:**

In this cohort, BAD and LAA imaging phenotypes were independently associated with END, whereas TyG was not independently associated. However, the direct discrimination advantage of the imaging model over the metabolic/coagulation model was not statistically established. The association with TT is exploratory, and the full model and nomogram require external validation before clinical use.

## Introduction

Acute isolated pontine infarction (IPI) is a clinically important form of posterior circulation ischemic stroke. In a recent prospective cohort of 408 patients with acute IPI, early neurological deterioration (END) occurred in 120 patients (29.4%) (1), consistent with previous reports that END is common in this population and is associated with unfavorable functional outcomes (2, 3).

Several vascular and tissue-level processes have been proposed in END after IPI. BAD is commonly used to describe an infarction extending to the ventral pontine surface and may reflect disease at the origin of a penetrating branch (4). This imaging pattern differs from the small deep lesion typically attributed to small artery disease (SAD) and has been associated with larger infarct volume, more severe motor deficits, and higher rates of END (5–7). High-resolution MRI studies also suggest that parent-artery plaque may be present in some patients with these patterns (8, 9). Large artery atherosclerosis (LAA) involving the vertebrobasilar system may be associated with END through hemodynamic compromise, plaque progression, or artery-to-artery embolism (10, 11). In the present study, BAD, LAA, and SAD were treated as operational imaging phenotypes rather than definitive etiologic mechanisms.

Metabolic dysfunction has emerged as a potential predictor of END. Diabetes mellitus, chronic hyperglycemia, and insulin resistance are associated with endothelial dysfunction, oxidative stress, and impaired collateral circulation (12, 13). The triglyceride-glucose (TyG) index, a surrogate marker of insulin resistance, has shown prognostic value for END in acute ischemic stroke and single subcortical infarction (14–16). However, it remains unclear whether TyG retains independent predictive value after adjustment for imaging phenotype. Hemostatic abnormalities may also be associated with infarct progression, and previous studies have evaluated hemostatic activation markers in perforator-related stroke (17).

Therefore, we aimed to investigate clinical, imaging, metabolic, and coagulation predictors of END in patients with acute IPI and to establish an individualized risk prediction model.

## Materials and methods

### Study design and population

A total of 477 consecutive patients with acute posterior circulation infarction were admitted to the Department of Neurology, Xiaolan People's Hospital of ZhongShan, between March 2021 and April 2025. After exclusion of patients with infarction outside the pons, onset-to-admission time greater than 72 h, an adjudicated cardioembolic stroke, or incomplete screening data, 162 patients with acute IPI were included. The selection process is shown in Figure 1.

**Figure 1:**
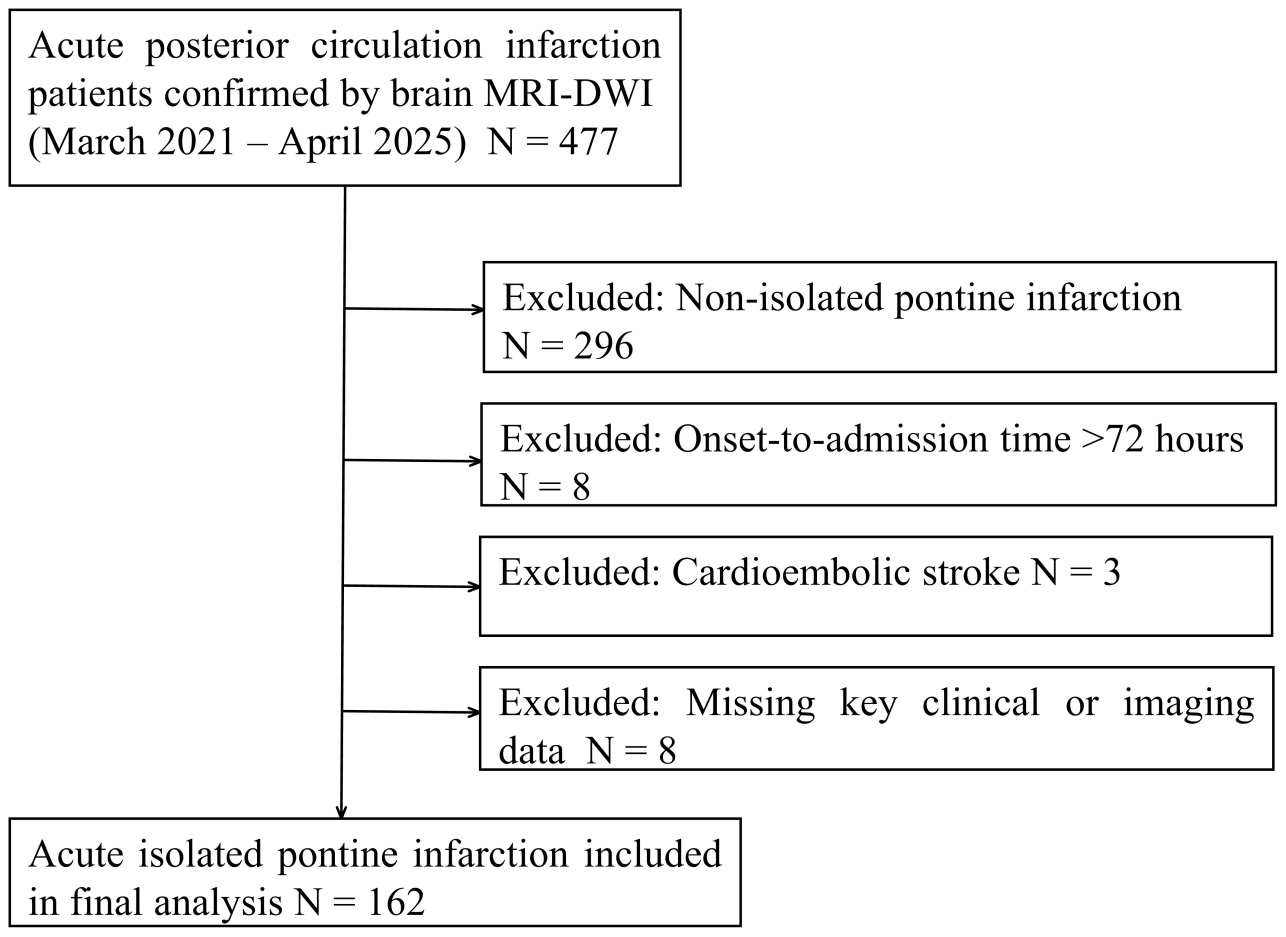
Flowchart of patient selection. Cardioembolic exclusion refers to an adjudicated cardioembolic index stroke and not to atrial fibrillation alone.

Inclusion criteria were: (1) age at least 18 years; (2) admission within 72 h of symptom onset, with neurological status assessed at admission and daily during the first 7 days after admission; and (3) acute infarction confined to the pons on diffusion-weighted imaging (DWI) obtained within 48 h of admission. Exclusion criteria were: (1) concurrent acute infarcts in other brain territories; (2) an index stroke adjudicated as cardioembolic after etiologic evaluation; (3) stroke attributed to arterial dissection, tumor, trauma, or another determined etiology; (4) pre-stroke modified Rankin Scale (mRS) score greater than 2; and (5) incomplete clinical, laboratory, or vascular imaging data required for analysis. Atrial fibrillation alone did not constitute automatic exclusion. Six patients with atrial fibrillation were retained because review of the source records documented that their index pontine strokes had not been adjudicated as cardioembolic. The study was approved by the Ethics Committee of Xiaolan People's Hospital of ZhongShan (approval No. 2024-0134). Informed consent was waived because anonymized data were analyzed retrospectively. The study complied with the Declaration of Helsinki.

### Imaging assessment

Brain MRI was performed within 48 h of admission using either a 3.0-T MAGNETOM Vida (Siemens Healthineers) or a 1.5-T SIGNA Infinity EchoSpeed (GE Healthcare) scanner. The standard protocol included axial diffusion-weighted imaging (DWI; *b* = 0 and 1,000 s/mm^2^), ADC, T2WI, and FLAIR sequences. On the 3.0-T system, DWI parameters were: TR/TE 6,980/80 ms, slice thickness 5.0 mm, gap 1.5 mm, and FOV 230 mm; on the 1.5-T system: TR/TE 4,871/90 ms, slice thickness 6.0 mm, gap 1.5 mm, and FOV 240 mm. Vascular assessment of the vertebrobasilar system was performed using 3D-TOF MRA (3.0-T: TR/TE 21/3.5 ms, 0.5-mm sections; 1.5-T: TR/TE 24/6.8 ms, 0.8-mm sections) or CTA (Philips Brilliance iCT; 0.8-mm sections, 100 kVp, 200 mAs) when clinically indicated. Two independent neuroradiologists, blinded to clinical data and outcomes, reviewed all images. The imaging phenotype was classified as BAD, LAA, or SAD based on ventral extension of the infarct and the degree of basilar or vertebral artery stenosis, with ≥50% stenosis defined as significant. Disagreements were resolved by consensus, and inter-rater reliability was assessed using Cohen's kappa coefficient (κ = 0.933).

### Data collection

The study collected baseline characteristics, including age, sex, time from symptom onset to admission, admission National Institutes of Health Stroke Scale (NIHSS) and modified Rankin Scale (mRS) scores, and documented comorbidities including hypertension, diabetes mellitus, coronary heart disease, atrial fibrillation, and leukoaraiosis. Standardized laboratory testing included blood cell counts, hemoglobin, fasting glucose, glycated hemoglobin (HbA1c), total cholesterol, triglycerides, high-density lipoprotein cholesterol, low-density lipoprotein cholesterol, urea nitrogen, creatinine, uric acid, albumin, fibrinogen, and thrombin time (TT). All MRI examinations, laboratory measurements, and baseline clinical variables used as candidate predictors were obtained before the first documented END event.

### Definitions

END was defined as an increase of at least 2 points in the total National Institutes of Health Stroke Scale (NIHSS) score or at least 1 point in the NIHSS motor subscore (item 5 or 6), relative to the admission baseline, during the first 7 days after admission (2). NIHSS was assessed at admission and daily throughout this 7-day window. The following indices were calculated: triglyceride-glucose (TyG) index = ln [triglycerides (mg/dl) x fasting glucose (mg/dl) / 2]; glucose-to-HbA1c ratio (GAR) = fasting glucose (mg/dl) / HbA1c (%); neutrophil-to-lymphocyte ratio (NLR) = neutrophil count / lymphocyte count; and monocyte-to-HDL ratio (MHR) = monocyte count / HDL-C (mmol/L). Patients were assigned to three operational imaging phenotypes rather than definitive etiologic mechanisms: branch atheromatous disease (BAD), defined as pontine infarction extending to the ventral surface without ≥50% stenosis; large artery atherosclerosis (LAA), defined as pontine infarction associated with at least 50% stenosis of the basilar or vertebral artery; and small artery disease (SAD), defined as a small deep pontine infarction not reaching the ventral surface and without significant vertebrobasilar stenosis (Supplementary Figure 1).

### Statistical analysis

Continuous variables are presented as mean ± standard deviation or median (interquartile range), as appropriate, and were compared using the independent-samples *t*-test or Mann–Whitney *U*-test. Categorical variables are presented as *n* (%) and were compared using the chi-square test or Fisher exact test. The final dataset included 162 patients with complete data for all 56 variables. Univariable logistic regression was used for exploratory screening. Because the full model included 49 END events and 7 coefficients and contained sparse outcome cells, Firth-penalized logistic regression was used as the primary multivariable method. Imaging phenotype was entered as a nominal factor with SAD as the reference, providing separate BAD- vs. -SAD and LAA-vs. -SAD estimates. Three cumulative association models were fitted: Model 1 included admission mRS and NIHSS; Model 2 additionally included imaging phenotype and diabetes; and Model 3 additionally included TyG and TT. Conventional maximum-likelihood regression was performed as a sensitivity analysis. Variance inflation factors were used to assess multicollinearity.

Discrimination was evaluated separately using four prespecified Firth models: base (mRS + NIHSS), base + imaging, base + metabolic/coagulation (diabetes + TyG + TT), and the full model. AUCs and 95% CIs were estimated using the DeLong method, with paired DeLong tests used for model comparisons. Percentile 95% CIs for ΔAUC were obtained from 2,000 paired bootstrap resamples with model refitting. The full Firth model underwent internal validation using 1,000 bootstrap resamples, from which optimism-corrected AUC, Brier score, calibration intercept, and calibration slope were calculated. Bootstrap-corrected calibration and decision curve analyses were considered exploratory. All tests were two-sided, with *P* < 0.05 considered statistically significant. All statistical procedures were executed in R (version 4.6.0).

## Results

### Baseline characteristics

Among 477 consecutive patients with acute posterior circulation infarction, 296 were excluded because the acute infarction was not confined to the pons. Among the remaining 181 patients, 8 were excluded for onset-to-admission time greater than 72 h, 3 for an adjudicated cardioembolic etiology, and 8 for incomplete screening data (3 missing HbA1c, 3 inadequate or missing vascular imaging, and 2 missing lipid measurements); these exclusion categories were mutually exclusive. The final cohort contained 162 patients and had no missing values across the 56 variables in the analytic dataset; no imputation was performed. Six patients with atrial fibrillation were retained after etiologic adjudication (3 with END and 3 without END). END occurred in 49 patients (30.2%) during the first 7 days after admission. The mean age was 60.22 ± 12.06 years, and 68.5% were men. Patients with END had longer hospital stays than those without END [12.00 (9.00–16.00) vs. 8.00 (6.00–10.00) days; *P* < 0.001]. Baseline NIHSS scores were slightly higher in the END group [4.00 (IQR 2.00–6.00) vs. 3.00 (IQR 2.00–5.00); *P* = 0.045]. Baseline characteristics are reported in Table 1.

**Table 1:** Clinical characteristics, laboratory findings, and in-hospital outcomes of patients with and without END.

Variable	Total (*n* = 162)	No END (*n* = 113)	END (*n* = 49)	*P*-value
Age, years	60.22 ± 12.06	59.81 ± 11.92	61.14 ± 12.46	0.530
Male sex	111 (68.5%)	81 (71.7%)	30 (61.2%)	0.260
Onset-to-admission time, h	24.00 (6.00, 48.00)	24.00 (7.00, 48.00)	12.00 (5.00, 24.00)	0.060
NIHSS score at admission	4.00 (2.00, 5.00)	3.00 (2.00, 5.00)	4.00 (2.00, 6.00)	0.045
mRS score at admission	4.00 (2.00, 4.00)	3.00 (2.00, 4.00)	4.00 (3.00, 4.00)	< 0.001
Hospital stay, days	8.00 (6.00, 12.75)	8.00 (6.00, 10.00)	12.00 (9.00, 16.00)	< 0.001
SBP, mmHg	161.75 ± 22.42	159.57 ± 22.08	166.80 ± 22.60	0.060
DBP, mmHg	93.81 ± 17.15	93.91 ± 17.71	93.59 ± 15.97	0.910
Hypertension	152 (93.8%)	109 (96.5%)	43 (87.8%)	0.070
Diabetes mellitus	77 (47.5%)	46 (40.7%)	31 (63.3%)	0.010
Coronary heart disease	6 (3.7%)	4 (3.5%)	2 (4.1%)	1.000
Atrial fibrillation	6 (3.7%)	3 (2.7%)	3 (6.1%)	0.370
Leukoaraiosis	141 (87.0%)	98 (86.7%)	43 (87.8%)	1.000
Pneumonia	21 (13.0%)	10 (8.8%)	11 (22.4%)	0.030
Imaging phenotype				< 0.001
BAD	39 (24.1%)	14 (12.4%)	25 (51.0%)	
LAA	53 (32.7%)	35 (31.0%)	18 (36.7%)	
SAD	70 (43.2%)	64 (56.6%)	6 (12.2%)	
WBC, × 10^9^/L	7.71 (6.51, 9.26)	7.70 (6.59, 9.30)	7.80 (6.10, 9.12)	0.670
Neutrophil count, × 10^9^/L	5.50 (4.34, 7.04)	5.34 (4.22, 7.38)	5.94 (4.42, 6.64)	0.660
Lymphocyte count, × 10^9^/L	1.51 (1.12, 2.04)	1.54 (1.14, 1.99)	1.46 (1.07, 2.06)	0.670
Monocyte count, × 10^9^/L	0.40 (0.31, 0.51)	0.41 (0.31, 0.52)	0.38 (0.30, 0.49)	0.320
Platelet count, × 10^9^/L	230.44 ± 58.28	234.68 ± 56.09	220.67 ± 62.55	0.180
Hemoglobin, g/L	138.46 ± 18.12	139.40 ± 18.70	136.31 ± 16.69	0.300
Glucose, mmol/L	6.97 (5.48, 10.04)	6.64 (5.32, 9.19)	7.63 (6.25, 13.45)	0.020
HbA1c, %	6.05 (5.50, 8.18)	5.90 (5.50, 7.32)	6.50 (5.70, 9.00)	0.020
Total cholesterol, mmol/L	4.89 ± 1.30	4.83 ± 1.24	5.02 ± 1.45	0.410
Triglycerides, mmol/L	1.54 (1.05, 2.20)	1.53 (1.02, 2.06)	1.59 (1.07, 2.77)	0.420
HDL-C, mmol/L	1.04 ± 0.25	1.05 ± 0.26	1.00 ± 0.23	0.170
LDL-C, mmol/L	3.25 ± 1.14	3.21 ± 1.05	3.32 ± 1.36	0.630
TyG index	9.08 (8.59, 9.74)	9.01 (8.58, 9.54)	9.44 (8.64, 9.98)	0.070
GAR	20.60 (17.01, 24.85)	19.46 (16.84, 23.43)	22.44 (18.44, 26.78)	0.050
NLR	3.28 (2.30, 5.77)	3.21 (2.23, 5.77)	3.60 (2.48, 5.77)	0.420
MHR	0.38 (0.28, 0.56)	0.38 (0.27, 0.57)	0.39 (0.28, 0.56)	0.830
Fibrinogen, g/L	3.17 (2.65, 3.74)	3.14 (2.64, 3.74)	3.28 (2.69, 3.68)	0.760
Thrombin time, s	19.50 (18.50, 20.80)	19.30 (18.50, 20.30)	20.00 (19.00, 21.40)	0.010
Urea nitrogen, mmol/L	5.01 (4.14, 6.29)	5.10 (4.30, 6.30)	4.66 (3.73, 6.17)	0.080
Creatinine, μmol/L	77.00 (65.05, 91.00)	79.00 (66.00, 91.45)	74.00 (64.40, 82.00)	0.110
Uric acid, μmol/L	345.00 (283.25, 417.85)	348.00 (284.00, 438.00)	339.00(274.60, 409.00)	0.450
Albumin, g/L	40.42 ± 3.91	40.60 ± 3.78	40.02 ± 4.21	0.410

Values are mean ± standard deviation, median (interquartile range), or *n* (%), as appropriate. BAD, branch atheromatous disease; DBP, diastolic blood pressure; GAR, glucose-to-HbA1c ratio; HbA1c, glycated hemoglobin; HDL-C, high-density lipoprotein cholesterol; LAA, large artery atherosclerosis associated with ≥50% stenosis of the basilar or vertebral artery; LDL-C, low-density lipoprotein cholesterol; MHR, monocyte-to-HDL ratio; mRS, modified Rankin Scale; NIHSS, National Institutes of Health Stroke Scale; NLR, neutrophil-to-lymphocyte ratio; SAD, small artery disease; SBP, systolic blood pressure; TyG, triglyceride-glucose index; WBC, white blood cell. Atrial fibrillation alone was not an exclusion criterion; six patients with atrial fibrillation were retained after the index stroke was adjudicated as noncardioembolic.

### Imaging phenotype and END

Imaging phenotype showed the largest between-group difference. BAD accounted for 51.0% of the END group and 12.4% of the non-END group, whereas SAD accounted for 12.2 and 56.6%, respectively; LAA accounted for 36.7 and 31.0%, respectively (*P* < 0.001) (Table 1). This distribution demonstrates an association between imaging phenotype and END but does not, by itself, establish a causal mechanism.

Inter-rater reliability for the three-category imaging classification was excellent. The unweighted Cohen's kappa was 0.933 (ASE: 0.025, *Z* = 37.88, *P* < 0.001), and the weighted kappa (squared weights) was 0.913 (ASE: 0.033, *Z* = 27.58, *P* < 0.001), indicating almost perfect agreement between the two independent readers. This finding supports the reproducibility of the imaging phenotype classification.

#### Metabolic and coagulation differences

Patients with END had higher glucose [7.63 (6.25–13.45) vs. 6.64 (5.32–9.19) mmol/L; *P* = 0.02] and higher HbA1c [6.50 (5.70–9.00) vs. 5.90 (5.50–7.32)%; *P* = 0.02]. The END group showed a higher rate of diabetes (63.3 vs. 40.7%; *P* = 0.01). TyG was higher in END patients but did not reach conventional significance in the baseline comparison [9.44 (8.64–9.98) vs. 9.01 (8.58–9.54); *P* = 0.07]. TT was significantly prolonged in the END group [20.00 (19.00–21.40) vs. 19.30 (18.50–20.30) seconds; *P* = 0.01].

### Univariable logistic regression

Univariable analysis identified associations of END with BAD, LAA, baseline mRS, glucose, HbA1c, TyG, TT, and diabetes. BAD had the largest univariable association (OR = 19.05, 95% CI 6.97–59.73; *P* < 0.001), followed by LAA (OR = 5.49, 95% CI 2.09–16.30; *P* < 0.001). TyG was associated with END before multivariable adjustment (OR = 1.51, 95% CI 1.02–2.26; *P* = 0.040). Among the exploratory calculated indices, GAR showed a borderline association (OR = 1.04, 95% CI 1.00–1.08; *P* = 0.080), whereas NLR (OR = 1.02, 95% CI 0.93–1.12; *P* = 0.674) and MHR (OR = 0.64, 95% CI 0.13–2.70; *P* = 0.552) were not associated with END (Supplementary Table 1).

### Multivariable logistic regression

In Firth Model 1, admission mRS was associated with END (OR = 1.75 per point, 95% CI 1.15–2.77; *P* = 0.009), whereas admission NIHSS was not (OR = 0.98, 95% CI 0.86–1.11; *P* = 0.697). After imaging phenotype and diabetes were added in Model 2, BAD (OR = 20.55, 95% CI 7.14–68.26; *P* < 0.001), LAA (OR = 4.83, 95% CI 1.80–14.36; *P* = 0.002), and diabetes (OR = 3.00, 95% CI 1.30–7.38; *P* = 0.010) were associated with END. In the fully adjusted Model 3, BAD (OR = 20.70, 95% CI 7.10–70.04; *P* < 0.001) and LAA (OR = 5.06, 95% CI 1.84–15.41; *P* = 0.001), each relative to SAD, remained associated with END. TT was also associated with END (OR = 1.34 per second, 95% CI 1.04–1.73; *P* = 0.020). Diabetes was borderline (OR = 2.62, 95% CI 0.97–7.46; *P* = 0.057), and TyG was not significant after adjustment (OR = 1.15, 95% CI 0.69–2.00; *P* = 0.603) (Table 2 and Figure 2).

**Table 2:** Firth-penalized multivariable logistic regression models for END.

Model	Variable	Adjusted OR	95% CI	*P*-value
Model 1: base clinical	Admission mRS, per 1-point increase	1.75	1.15–2.77	0.009
	Admission NIHSS, per 1-point increase	0.98	0.86–1.11	0.697
Model 2: Model 1 + imaging + diabetes	Admission mRS, per 1-point increase	1.49	0.93–2.51	0.095
	Admission NIHSS, per 1-point increase	0.92	0.79–1.05	0.225
	Imaging: BAD vs. SAD	20.55	7.14–68.26	< 0.001
	Imaging: LAA vs. SAD	4.83	1.80–14.36	0.002
	Diabetes mellitus: yes vs. no	3.00	1.30–7.38	0.010
Model 3: full (Model 2 + TyG + TT)	Admission mRS, per 1-point increase	1.37	0.85–2.32	0.199
	Admission NIHSS, per 1-point increase	0.95	0.81–1.10	0.533
	Imaging: BAD vs. SAD	20.70	7.10–70.04	< 0.001
	Imaging: LAA vs. SAD	5.06	1.84–15.41	0.001
	Diabetes mellitus: yes vs. no	2.62	0.97–7.46	0.057
	TyG, per 1-unit increase	1.15	0.69–2.00	0.603
	TT, per 1-second increase	1.34	1.04–1.73	0.020

Models are cumulative. Imaging phenotype was modeled as a nominal factor with SAD as the reference, yielding separate BAD-vs.-SAD and LAA-vs.-SAD estimates; no ordinal trend was assumed. Odds ratios, profile penalized-likelihood 95% CIs, and P-values were obtained from Firth-penalized logistic regression. BAD, branch atheromatous disease; CI, confidence interval; LAA, large artery atherosclerosis; mRS, modified Rankin Scale; NIHSS, National Institutes of Health Stroke Scale; OR, odds ratio; SAD, small artery disease; TT, thrombin time; TyG, triglyceride-glucose index. The association models in this table are distinct from the four discrimination models in Table 3.

**Figure 2:**
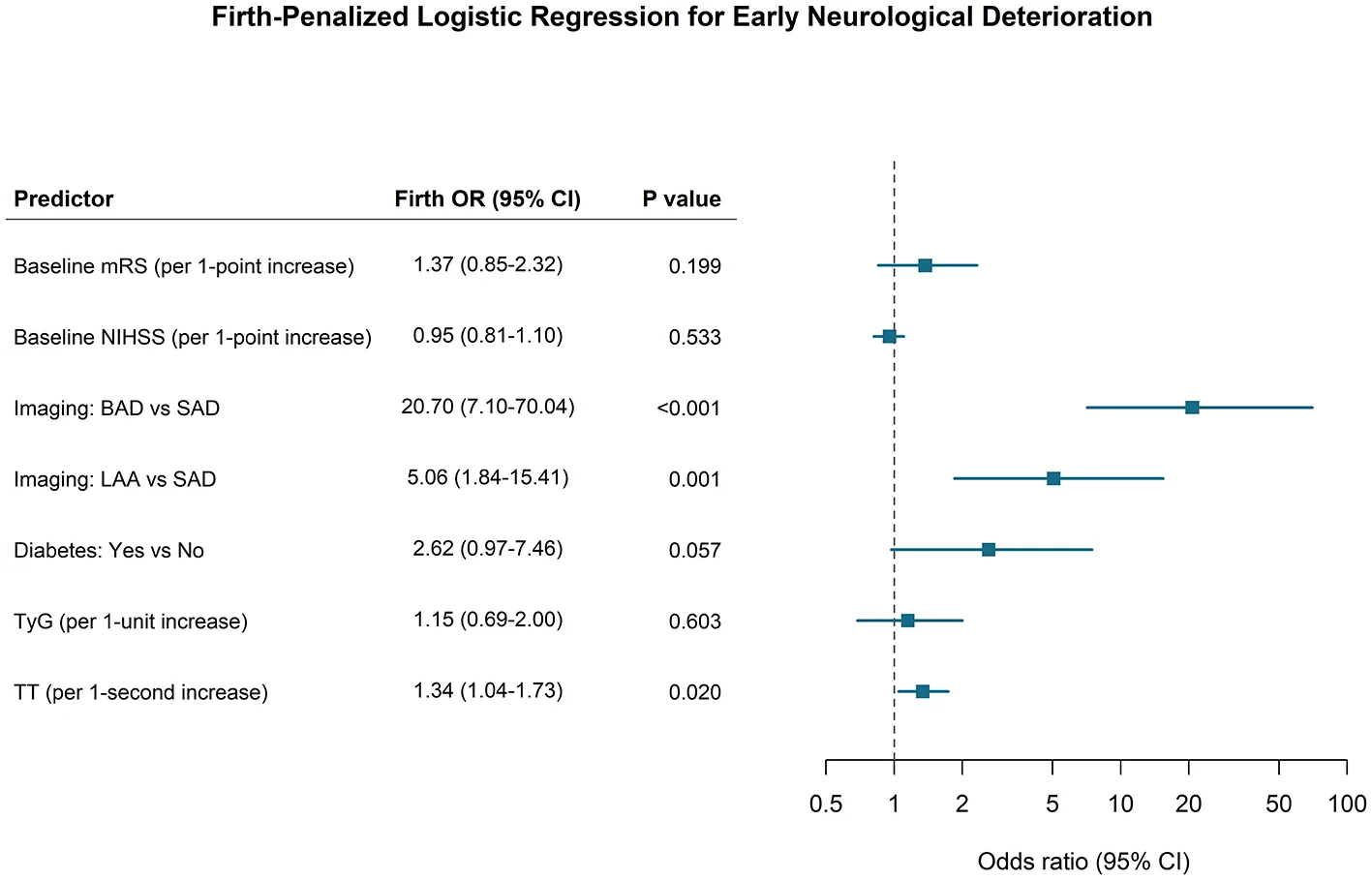
Forest plot of the primary Firth-penalized multivariable logistic regression model (Model 3) for END. Imaging phenotype was modeled as a nominal factor with SAD as the reference. OR, odds ratio; CI, confidence interval; BAD, branch atheromatous disease; LAA, large artery atherosclerosis (at least 50% stenosis of the basilar or vertebral artery); SAD, small artery disease; TyG, triglyceride-glucose index; TT, thrombin time; mRS, modified Rankin Scale; NIHSS, National Institutes of Health Stroke Scale.

Variance inflation factors (VIFs) for Model 3 ranged from 1.04 to 1.64 (Supplementary Table 2), below the prespecified threshold of 5. VIFs for the imaging contrasts, diabetes, and TyG were 1.60–1.64, 1.47, and 1.35, respectively, and did not indicate problematic collinearity.

### Conventional maximum-likelihood sensitivity analysis

Conventional maximum-likelihood estimates were directionally consistent with the primary Firth estimates: BAD vs. SAD OR 25.70 (95% CI 8.34–93.32), LAA vs. SAD OR 5.67 (95% CI 1.98–18.31), diabetes OR 2.77 (95% CI 0.99–8.27), and TT OR 1.37 (95% CI 1.06–1.79). The side-by-side estimates are reported in Supplementary Table 3; statistical inference in the main text and Table 2 is based on Firth regression.

### Predictive performance and nomogram

The apparent AUCs were 0.652 (95% CI 0.562–0.742) for the base model, 0.814 (95% CI 0.745–0.883) for base plus imaging, 0.716 (95% CI 0.631–0.802) for base plus metabolic/coagulation markers, and 0.852 (95% CI 0.792–0.911) for the full model (Table 3 and Supplementary Figure 2). Relative to the base model, the AUC increase was 0.162 for imaging (bootstrap 95% CI 0.069–0.260; paired DeLong *P* = 0.001) and 0.064 for the metabolic/coagulation block (bootstrap 95% CI 0.012–0.158; paired DeLong *P* = 0.080). The direct comparison between these two augmented models did not establish superior discrimination for imaging (Delta AUC = 0.098, bootstrap 95% CI −0.015–0.180; paired DeLong *P* = 0.066). For descriptive completeness, the apparent full-model Youden cutoff was 0.236, with 85.7% sensitivity and 69.0% specificity; this was not treated as a validated clinical threshold.

**Table 3:** Apparent discrimination of four prespecified Firth-penalized models for END.

Model	Predictors	Apparent AUC (95% CI)	ΔAUC vs. base (bootstrap 95% CI)	Paired DeLong *P* vs. base
Base	mRS + NIHSS	0.652 (0.562–0.742)	Reference	–
Base + imaging	mRS + NIHSS + imaging	0.814 (0.745–0.883)	0.162 (0.069–0.260)	0.001
Base + metabolic/coagulation	mRS + NIHSS + diabetes + TyG + TT	0.716 (0.631–0.802)	0.064 (0.012–0.158)	0.080
Full	All predictors	0.852 (0.792–0.911)	0.199 (0.117–0.305)	< 0.001

These discrimination models were specified separately from the cumulative association models in Table 2. AUC, area under the curve; CI, confidence interval; END, early neurological deterioration; mRS, modified Rankin Scale; NIHSS, National Institutes of Health Stroke Scale; TT, thrombin time; TyG, triglyceride-glucose index.

All 1,000 bootstrap iterations of the same Firth model succeeded. The apparent and optimism-corrected AUCs were 0.852 and 0.818, respectively. The ROC curve for the full model is shown in Figure 3. The optimism-corrected Brier score was 0.162, the calibration intercept was −0.023, and the calibration slope was 0.870 (Supplementary Figure 3). The exploratory bootstrap-corrected DCA is shown in Supplementary Figure 4.

**Figure 3:**
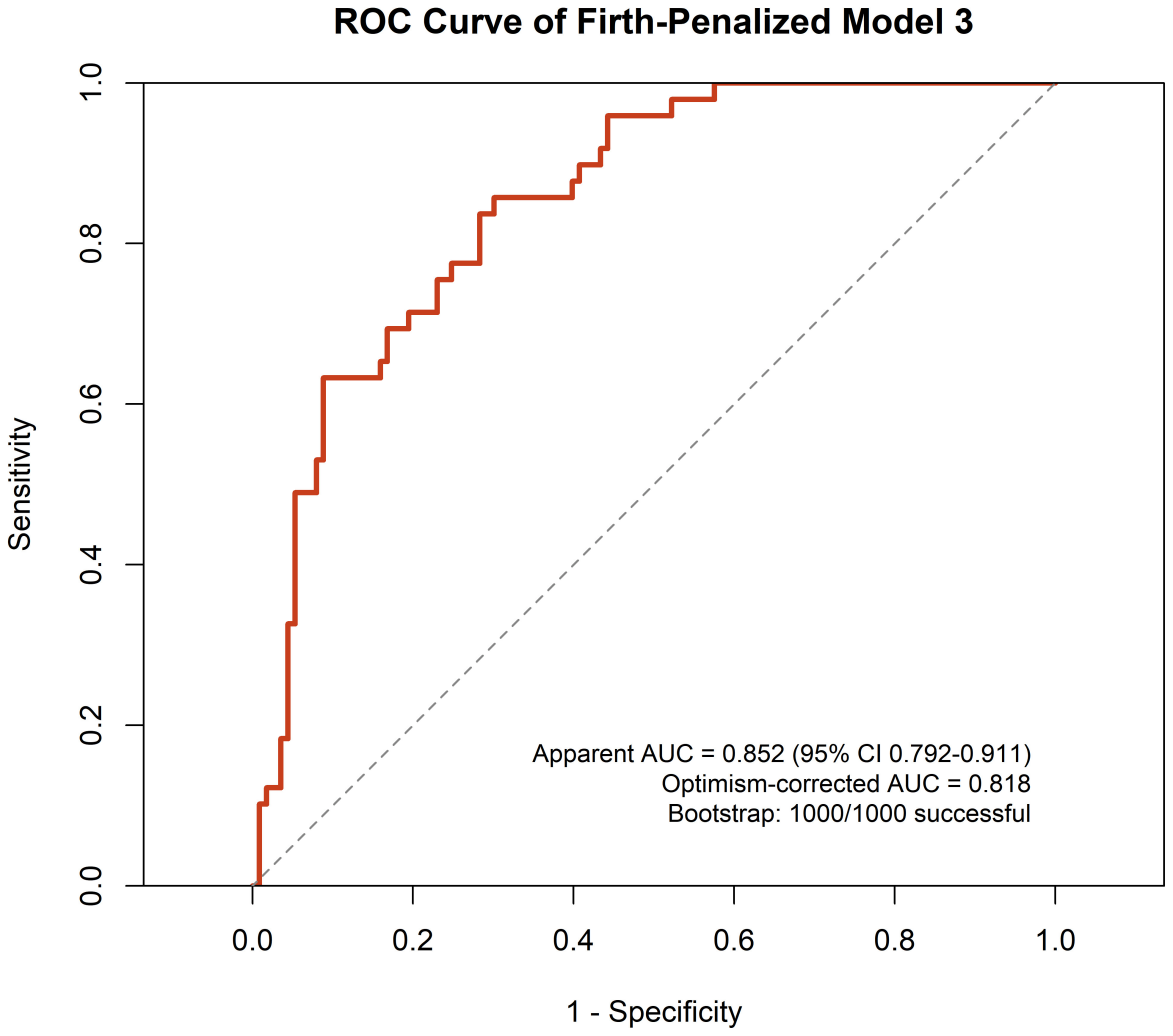
Receiver operating characteristic curve of the full Firth model for END. The apparent AUC was 0.852 (95% CI 0.792-0.911). AUC, area under the curve.

An exploratory nomogram was derived from the full Firth model (Figure 4). Because no independent external cohort was available, neither the nomogram nor the DCA was interpreted as evidence of clinical utility or readiness for implementation.

**Figure 4:**
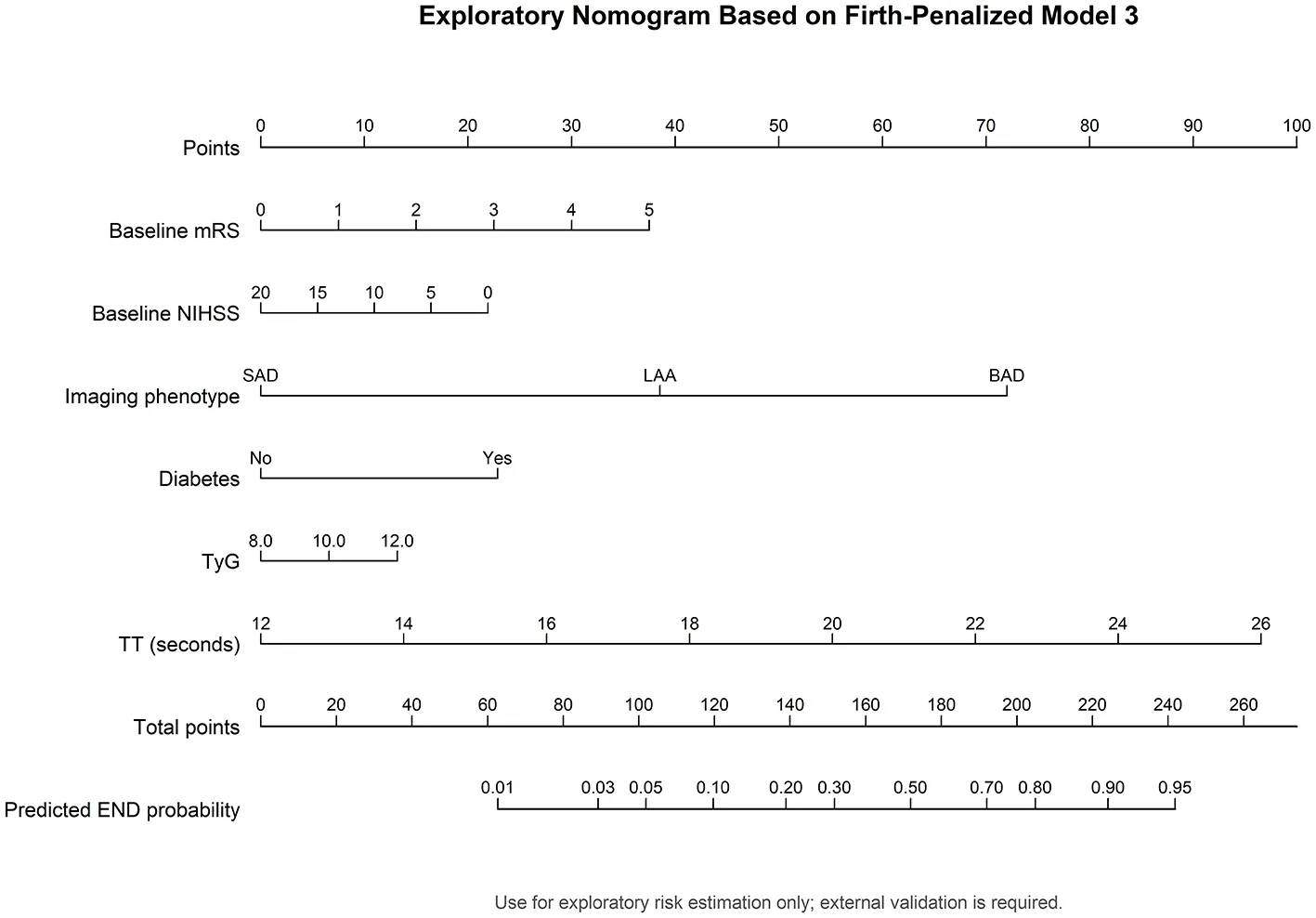
Exploratory nomogram derived from the full Firth model for estimating END probability after acute IPI. The nomogram has not undergone external validation and is not intended to define a treatment threshold. mRS, modified Rankin Scale; NIHSS, National Institutes of Health Stroke Scale; TyG, triglyceride-glucose index; TT, thrombin time; END, early neurological deterioration.

## Discussion

In this retrospective cohort of patients with acute IPI, 30.2% developed END. In the primary Firth analysis, BAD and LAA imaging phenotypes were associated with substantially higher odds of END than SAD, although the wide confidence intervals indicate considerable uncertainty in effect magnitude. TT also remained associated with END, diabetes showed borderline evidence of association, and TyG was not independently associated after adjustment. Adding imaging to the base model produced a larger observed AUC increase than adding the metabolic/coagulation block, but the direct difference between these two augmented models was not statistically significant. Thus, although imaging produced the larger observed increment in discrimination, its superiority over the metabolic/coagulation model was not statistically established.

END is a clinically important complication of pontine infarction, occurring in approximately 20%−30% of patients in previous cohorts (1–3, 18). The clinical significance of END lies in its strong association with prolonged hospitalization, increased disability, and reduced likelihood of functional independence. In line with these observations, our study found that patients who developed END had significantly longer hospital stays and higher baseline mRS scores compared to those without END. Our observed END rate of 30.2% was consistent with previous IPI cohorts (1–3, 18, 19). The pathophysiological mechanisms of END are multifactorial, involving infarct expansion, hemodynamic compromise, thrombus propagation, and inflammatory activation (20). However, the relative contribution of each mechanism has remained incompletely understood, particularly in the context of isolated pontine infarction, where the compact anatomy of the brainstem amplifies the clinical impact of even small lesion changes.

The BAD imaging phenotype had the largest adjusted association with END (Firth OR = 20.70, 95% CI 7.10–70.04). This observation is consistent with reports that pontine infarcts extending to the ventral surface are prone to neurological progression (5, 21, 22). One plausible explanation is the involvement of the proximal perforator origin and subsequent infarct expansion; however, BAD, LAA, and SAD in this study were operational imaging phenotypes rather than definitive pathologic mechanisms. The compact organization of corticospinal fibers in the ventral pons may magnify the clinical consequences of relatively small increments in ischemic injury (23, 24). The association observed for LAA is compatible with parent-artery atherosclerosis and involvement of basilar perforator origins, as suggested by high-resolution MRI studies (11, 25). These mechanisms were not directly measured, and the present data support association rather than causation.

In contrast to previous studies reporting associations between TyG and END in broader ischemic stroke or subcortical infarction populations (14–16), TyG was not independently associated with END in our full model. Diabetes showed borderline evidence of association. These findings do not establish that metabolic effects are mediated through BAD or LAA; such a pathway remains hypothetical and would require longitudinal data and formal mediation analysis.

We additionally identified TT as a variable independently associated with END. This finding should be interpreted as exploratory because TT is an *in vitro* screening assay that measures the time required for fibrin formation after exogenous thrombin is added to plasma. It is influenced by fibrinogen concentration or function and may be prolonged by fibrin degradation products, heparin, direct thrombin inhibitors, and severe liver dysfunction (26). Therefore, TT cannot be interpreted as a direct measure of *in vivo* thrombin activation or local thrombus formation. Although TT remained associated with END after adjustment, this finding is exploratory and does not establish a thrombotic mechanism or support treatment selection. Prospective studies using standardized sampling and activation-specific hemostatic biomarkers are required (17, 26).

The full model had an apparent AUC of 0.852 (95% CI 0.792–0.911) and an optimism-corrected AUC of 0.818 after 1,000 successful bootstrap iterations. The optimism-corrected calibration intercept was −0.023, the calibration slope was 0.870, and the Brier score was 0.162. These results indicate good apparent discrimination but also measurable optimism and calibration shrinkage. Accordingly, the nomogram and DCA are exploratory and should not be used for clinical decision-making before external validation.

Recognition of the BAD imaging phenotype may have implications for the intensity of clinical surveillance rather than for phenotype-specific treatment. Patients with this pattern may warrant closer neurological monitoring during early hospitalization and prompt diagnostic reassessment, including repeat parenchymal or vascular imaging when clinically indicated. However, imaging phenotype should not supersede established guideline-based eligibility criteria for reperfusion or antiplatelet therapy. The 2026 AHA/ASA guideline does not provide a BAD-specific treatment pathway (27). Although several antithrombotic strategies have been investigated in BAD, no phenotype-specific treatment has established efficacy (6, 10). Prospective multicenter studies and randomized trials are required to determine whether treatment tailored to imaging phenotype improves clinical outcomes.

### Limitations

Several limitations should be considered. First, this retrospective single-center study is susceptible to selection bias and residual confounding. Second, there were 49 END events for 7 model coefficients, and the SAD-END cell contained only 6 patients; Firth penalization reduces but does not eliminate the resulting uncertainty, as reflected by the wide confidence intervals. Third, the BAD/LAA/SAD categories were operational imaging phenotypes and should not be equated with definitive etiologic mechanisms. Fourth, TT was measured at a single time point and is influenced by multiple laboratory and clinical factors; the observed association is exploratory. Fifth, although every candidate predictor was documented before the first END event and all 1,000 bootstrap iterations succeeded, internal validation cannot establish transportability. No independent external validation cohort was available. The nomogram, calibration plot, and DCA should therefore be regarded as hypothesis-generating pending prospective multicenter external validation.

## Conclusion

In conclusion, BAD and LAA imaging phenotypes were strongly associated with END after acute IPI, whereas TyG was not independently associated in the full Firth model. TT was also associated with END, but this finding does not establish *in vivo* thrombin activation or a treatment target. The exploratory full model showed optimism-corrected discrimination but requires external validation before any clinical application.

## Data Availability

The original contributions presented in the study are included in the article/Supplementary material, further inquiries can be directed to the corresponding authors.
