# Supplementary material for: Imaging phenotype is more strongly associated with early neurological deterioration after acute isolated pontine infarction: a retrospective cohort study
Source: Front Neurol. 2026 Oct 8;17:1913826. doi: 10.3389/fneur.2026.1913826 (PMC13686867; doi:10.3389/fneur.2026.1913826)
Supplement: Supplementary file 1 [file Table_1.docx]

**Supplemental Table 1. Univariable conventional maximum-likelihood logistic regression analysis of END.**

| **Variable** | **OR** | **95% CI** | **P value** |
| --- | --- | --- | --- |
| Imaging: BAD vs SAD | 19.05 | 6.97-59.73 | <0.001 |
| Imaging: LAA vs SAD | 5.49 | 2.09-16.30 | <0.001 |
| Admission mRS, per 1-point increase | 1.69 | 1.19-2.48 | 0.005 |
| Admission NIHSS, per 1-point increase | 1.08 | 0.97-1.20 | 0.168 |
| Age, per 1-year increase | 1.01 | 0.98-1.04 | 0.519 |
| Male vs female | 0.62 | 0.31-1.27 | 0.190 |
| Onset-to-admission time, per 1-hour increase | 0.99 | 0.97-1.00 | 0.084 |
| SBP, per 1-mmHg increase | 1.01 | 1.00-1.03 | 0.061 |
| DBP, per 1-mmHg increase | 1.00 | 0.98-1.02 | 0.913 |
| Hypertension: yes vs no | 0.26 | 0.06-0.97 | 0.046 |
| Diabetes mellitus: yes vs no | 2.51 | 1.27-5.08 | 0.009 |
| Coronary heart disease: yes vs no | 1.16 | 0.16-6.15 | 0.867 |
| Atrial fibrillation: yes vs no | 2.39 | 0.43-13.34 | 0.297 |
| Leukoaraiosis: yes vs no | 1.10 | 0.41-3.25 | 0.858 |
| Glucose, per 1-mmol/L increase | 1.09 | 1.02-1.16 | 0.009 |
| HbA1c, per 1-percentage-point increase | 1.17 | 1.01-1.35 | 0.031 |
| Triglycerides, per 1-mmol/L increase | 1.15 | 0.94-1.47 | 0.197 |
| HDL-C, per 1-mmol/L increase | 0.40 | 0.10-1.54 | 0.192 |
| LDL-C, per 1-mmol/L increase | 1.08 | 0.81-1.45 | 0.595 |
| TyG, per 1-unit increase | 1.51 | 1.02-2.26 | 0.040 |
| GAR, per 1-unit increase | 1.04 | 1.00-1.08 | 0.080 |
| WBC, per 1 × 10⁹/L increase | 0.96 | 0.84-1.09 | 0.572 |
| Neutrophil count, per 1 × 10⁹/L increase | 1.05 | 0.96-1.16 | 0.319 |
| Lymphocyte count, per 1 × 10⁹/L increase | 1.00 | 0.62-1.60 | 0.992 |
| Monocyte count, per 1 × 10⁹/L increase | 0.35 | 0.04-2.38 | 0.297 |
| Platelet count, per 1 × 10⁹/L increase | 1.00 | 0.99-1.00 | 0.161 |
| Hemoglobin, per 1-g/L increase | 0.99 | 0.97-1.01 | 0.318 |
| NLR, per 1-unit increase | 1.02 | 0.93-1.12 | 0.674 |
| MHR, per 1-unit increase | 0.64 | 0.13-2.70 | 0.552 |
| Fibrinogen, per 1-g/L increase | 0.92 | 0.64-1.15 | 0.572 |
| TT, per 1-second increase | 1.35 | 1.09-1.70 | 0.009 |
| Urea nitrogen, per 1-mmol/L increase | 0.82 | 0.66-1.00 | 0.065 |
| Creatinine, per 1-μmol/L increase | 0.99 | 0.97-1.00 | 0.108 |
| Uric acid, per 1-μmol/L increase | 1.00 | 0.99-1.00 | 0.296 |
| Albumin, per 1-g/L increase | 0.96 | 0.88-1.05 | 0.388 |

**Note:** Odds ratios, profile-likelihood 95% CIs, and P values in this screening table were estimated using conventional maximum-likelihood logistic regression; the primary multivariable inference used Firth-penalized regression. Imaging was modeled nominally with SAD as the reference. BAD, branch atheromatous disease; CI, confidence interval; DBP, diastolic blood pressure; GAR, glucose-to-HbA1c ratio; HbA1c, glycated hemoglobin; HDL-C, high-density lipoprotein cholesterol; LAA, large artery atherosclerosis associated with ≥50% stenosis of the basilar or vertebral artery; LDL-C, low-density lipoprotein cholesterol; MHR, monocyte-to-HDL ratio; mRS, modified Rankin Scale; NIHSS, National Institutes of Health Stroke Scale; NLR, neutrophil-to-lymphocyte ratio; OR, odds ratio; SAD, small artery disease; SBP, systolic blood pressure; TT, thrombin time; TyG, triglyceride-glucose index; WBC, white blood cell.
