# Supplementary material for: Imaging phenotype is more strongly associated with early neurological deterioration after acute isolated pontine infarction: a retrospective cohort study
Source: Front Neurol. 2026 Oct 8;17:1913826. doi: 10.3389/fneur.2026.1913826 (PMC13686867; doi:10.3389/fneur.2026.1913826)
Supplement: Supplementary file 2 [file Table_2.docx]

**Supplemental Table 2. Variance inflation factors for variables in the full Model 3.**

| **Variable** | **VIF** | **Interpretation** |
| --- | --- | --- |
| Admission mRS | 1.48 | No concerning multicollinearity |
| Admission NIHSS | 1.51 | No concerning multicollinearity |
| Imaging: BAD vs SAD | 1.64 | No concerning multicollinearity |
| Imaging: LAA vs SAD | 1.60 | No concerning multicollinearity |
| Diabetes mellitus: yes vs no | 1.47 | No concerning multicollinearity |
| TyG | 1.35 | No concerning multicollinearity |
| TT | 1.04 | No concerning multicollinearity |

**Note:** VIFs below the prespecified threshold of 5 were interpreted as not indicating concerning multicollinearity. Imaging was represented by separate BAD-versus-SAD and LAA-versus-SAD dummy variables. BAD, branch atheromatous disease; LAA, large artery atherosclerosis associated with ≥50% stenosis of the basilar or vertebral artery; mRS, modified Rankin Scale; NIHSS, National Institutes of Health Stroke Scale; SAD, small artery disease; TT, thrombin time; TyG, triglyceride-glucose index; VIF, variance inflation factor.
