# Supplementary material for: Imaging phenotype is more strongly associated with early neurological deterioration after acute isolated pontine infarction: a retrospective cohort study
Source: Front Neurol. 2026 Oct 8;17:1913826. doi: 10.3389/fneur.2026.1913826 (PMC13686867; doi:10.3389/fneur.2026.1913826)
Supplement: Supplementary file 3 [file Table_3.docx]

**Supplemental Table 3. Sensitivity analysis comparing conventional maximum-likelihood and Firth-penalized estimates for the full Model 3.**

| **Variable** | **ML OR (95% CI)** | **ML P value** | **Firth OR (95% CI)** | **Firth P value** |
| --- | --- | --- | --- | --- |
| Admission mRS, per 1-point increase | 1.42 (0.86-2.47) | 0.182 | 1.37 (0.85-2.32) | 0.199 |
| Admission NIHSS, per 1-point increase | 0.94 (0.80-1.10) | 0.480 | 0.95 (0.81-1.10) | 0.533 |
| Imaging: BAD vs SAD | 25.70 (8.34-93.32) | <0.001 | 20.70 (7.10-70.04) | <0.001 |
| Imaging: LAA vs SAD | 5.67 (1.98-18.31) | 0.002 | 5.06 (1.84-15.41) | 0.001 |
| Diabetes mellitus: yes vs no | 2.77 (0.99-8.27) | 0.058 | 2.62 (0.97-7.46) | 0.057 |
| TyG, per 1-unit increase | 1.18 (0.69-2.09) | 0.563 | 1.15 (0.69-2.00) | 0.603 |
| TT, per 1-second increase | 1.37 (1.06-1.79) | 0.019 | 1.34 (1.04-1.73) | 0.020 |

**Note:** The two analyses used the identical Model 3 specification: admission mRS, admission NIHSS, nominal imaging phenotype, diabetes, TyG, and TT. Imaging was modeled as an unordered factor with SAD as the reference, and BAD and LAA were estimated separately. Firth-penalized regression was the primary analysis; conventional maximum-likelihood estimates are shown only as a sensitivity analysis. BAD, branch atheromatous disease; CI, confidence interval; LAA, large artery atherosclerosis; ML, maximum likelihood; mRS, modified Rankin Scale; NIHSS, National Institutes of Health Stroke Scale; OR, odds ratio; SAD, small artery disease; TT, thrombin time; TyG, triglyceride-glucose index.
